# Effects of continuous irrigation at room temperature or +4ºC on the cyclic fatigue resistance of K3XF instruments

**DOI:** 10.34172/joddd.2020.038

**Published:** 2020-09-21

**Authors:** Hakan Arslan, Ezgi Doğanay Yıldız, Gizem Taş, Ertuğrul Karataş, Ebru Tepecik

**Affiliations:** ^1^Department of Endodontics, Faculty of Dentistry, Health Sciences University, İstanbul, Turkey; ^2^Department of Endodontics, Faculty of Dentistry, Bursa Uludağ University, Bursa, Turkey; ^3^Çorlu Oral and Dental Health Hospital, Tekirdağ, Turkey; ^4^Department of Endodontics, Faculty of Dentistry, Ataturk University, Erzurum, Turkey; ^5^Private Practice, Dental Clinic, İstanbul, Turkey

**Keywords:** Cyclic Fatigue, Irrigation, K3XF

## Abstract

**Background.** The present study aimed to evaluate the impact of continuous irrigation with saline solution at room temperature or +4°C on the cyclic fatigue resistance of K3XF files.

**Methods.** Forty-eight new K3XF files (#30, .04 taper) were randomly assigned to three groups: control group (no irrigation), continuous irrigation with saline solution at room temperature, and continuous irrigation with saline solution at +4°C. The instruments were tested in an artificial, stainless steel root canal with a double curvature at body temperature (37±1°C). Time to fracture was converted to the number of cycles to fracture (NCF). The lengths of the fractured fragments were recorded. Kruskal–Wallis H test and one-way ANOVA were used to analyze data.

**Results.** K3XF files’ cyclic fatigue resistance was significantly higher in the continuous irrigation groups than in the control group. Continuous irrigation with saline solution at +4°C resulted in higher cyclic fatigue resistance than continuous irrigation with saline solution at room temperature. There were no significant differences between the groups in terms of the fractured fragments’ length.

**Conclusion.** Within this study’s limitations, continuous irrigation with saline solution increased the NCF of NiTi instruments; decreasing the saline solution’s temperature increased this effect.

## Introduction

One of the main drawbacks of nickel-titanium (NiTi) files is file fracture during use in root canals.^1,2^ Fracture of NiTi rotary files occurs due to two reasons, i.e., cyclic and torsional fatigue, have been described.^3,4^ Fracture because of cyclic fatigue is associated with rotation in a curved root canal, which causes cyclic tension and compression, leading to micro-crack formation and, ultimately, file separation.^3,5,6^ The mechanism of file fracture is multifactorial and complex, and cyclic fatigue resistance of NiTi files is affected by many factors, such as instrument characteristics, the operator, operation speed, and root canal anatomy.^2,7^

Manufacturers aim to increase files’ fatigue life; therefore, this has prompted the manufacturers to develop new NiTi rotary instruments to enhance the mechanical characteristics through innovative design and manufacturing processes.^8,9^ Thermal treatment of NiTi alloys, such as M-Wire, R-phase wire, and controlled-memory wire, has been tried to optimize the mechanical properties of files.^10-13^ The relative proportions and features of the microstructural phases affect NiTi alloy’s mechanical properties.^8^ K3XF (SybronEndo, Orange, CA) is a file produced from the R-phase wire. The R-phase provides shape memory and good superelasticity effects; it has lower Young’s modulus than the austenite phase. This means that a file produced from the R-phase wire would be less rigid.^14^

Phase transformation temperatures of NiTi instruments affect the mechanical behavior of instruments.^15^ The NiTi alloy in the martensitic phase is more ductile than in the austenitic phase. Recent studies have demonstrated that more martensitic files have a longer fatigue life than austenitic files.^16,17^ Continuous irrigation might delay austenite phase transformation. However, according to the current literature, there are no studies on the effect of continuous irrigation with saline at room temperature or +4°C on the cyclic fatigue resistance of files. The null hypothesis was that there would be no significant difference between the groups.

## Methods

### Pilot study

Before the main study, a pilot study was carried out using 15 K3XF instruments (#30, 0.04 taper). Files were selected for the study after confirming that there was no visible defect or irregularity at ×20 magnification under a stereomicroscope (Novex, Arnhem, Holland).

**Figure 1:**
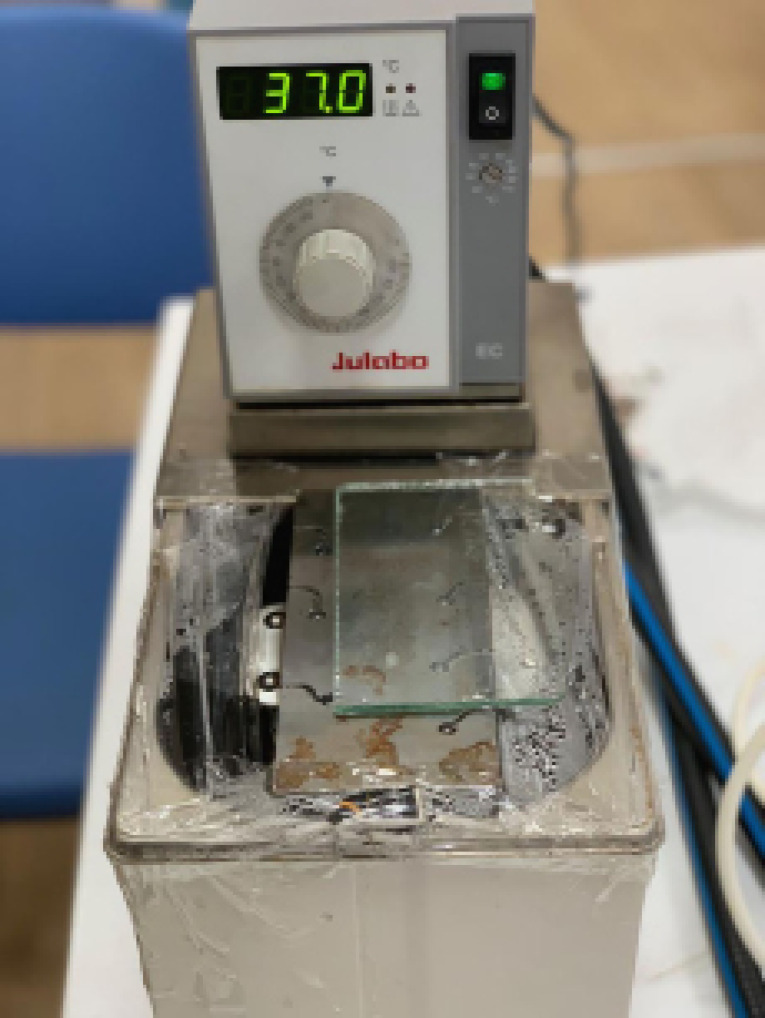
Experimental design.

The files were rotated within a tapered artificial root canal with a coronal and an apical curvature (double curvature). The coronal curvature’s angle was 60°, and its radius was 5 mm. The apical curvature’s angle was 70°, and its radius was 2 mm. The artificial root canal’s length was 21 mm. The files were rotated using a torque-controlled endodontic motor (VDW Silver; VDW, Munich, Germany) according to the manufacturer’s instructions at 350 rpm and 150 g-cm torque. The handpiece was fixed during the test. The curvatures were located 7 and 12 mm from the tip of the instrument. The test was performed in a water bath (Julabo, Seelbach, Germany) at 37±1°C.^18^ Cyclic fatigue test was started after the artificial canal temperature was confirmed using a thermocouple (Figure 1). Instruments’ slipping out was prevented by covering the artificial canal with glass.

### Pilot study

The instruments were randomly divided into three groups (n=5) using a web page (http://www.randomizer.org):

**Control group:** K3XF instruments were used without any irrigation.

**Continuous irrigation with saline solution at room temperature:** K3XF instruments were operated under continuous irrigation with saline solution at room temperature.

**Continuous irrigation with saline solution at +4°C:**K3XF instruments were operated under continuous irrigation with saline solution at +4°C. Saline was placed in a refrigerator for one day. In this group, the injectors were also placed in the refrigerator. During the experiment, to prevent the saline solution from heating, the injectors were stored in a container with dry ice (Elabscience Biotechnology Co. Ltd, Wuhan, China).

In the irrigation groups, the irrigation needle was placed in the coronal part of the stainless steel canal, and irrigation was performed at a constant speed of 10 mL/min. The time when the file was fractured was recorded in seconds. Time to fracture was converted to the number of cycles to fracture (NCF). The fractured fragments’ length was measured and recorded in millimeters.

### Main study

The data obtained from the pilot study were analyzed; the effect size was 17.394. According to the power analysis, six samples were sufficient, with a power of 0.95. However, a worst-case scenario approach was used; therefore, the actual sample size was 48 instruments (n=16). Thirty-three more instruments were added to the original 15, and they were also randomly assigned to the three experimental groups.

### Statistical analysis

NCF data were analyzed using the Kruskal–Wallis H test; fractured fragments’ length data were analyzed with one-way ANOVA. All statistical analyses were performed with SPSS 20 for IOS (SPSS Inc., Chicago, IL, USA).The statistical significance level was set at 5%.

## Results

Table 1 presents the means and standard deviations of NCF and fractured fragments’ lengths. K3XF instruments’ cyclic fatigue resistance was significantly higher in the continuous irrigation groups than in the control group (P<0.05). Continuous irrigation with saline solution at +4°C resulted in higher cyclic fatigue resistance than continuous irrigation with saline solution at room temperature (P<0.05). There were no significant differences (P>0.05) between the groups in terms of the fractured fragments’ length.

**Table 1:** The means and standard deviations for NCF and fractured fragments’ lengths (mm)

**Groups**	**NCF ± SD**	**Length of the Fractured Fragment** **(mm) ± SD**
**Control group**	281.45 ± 38.25^a^	12.53 ± 0.38^a^
**Continuous irrigation with saline solution at room temperature**	564.01 ± 66.73^b^	12.28 ± 0.31^a^
**Continuous irrigation with saline solution at +4°C**	818.48 ± 139.41^c^	12.31 ± 0.81^a^

SD, standard deviation. Different superscript lowercase letters in the same column indicate a mean significant difference (P<0.05).

## Discussion

Cyclic fatigue failure occurs because of procedural difficulty related to repeated extension and compression of files while rotating in a curved canal.^5,6^ It has been shown that growth rates of fatigue cracks in NiTi alloys are significantly higher than other metals with similar strength.^19^ Thus, when a microcrack occurs, it can rapidly lead to instrument fracture. This is probably the main reason for many broken instruments associated with fatigue failure seen clinically.^3,4,20^ The present study investigated the effect of cooling the instruments by continuous irrigation with saline solution at room temperature or +4°C on cyclic fatigue resistance. The experiment was performed in a 37°C water bath to simulate the body temperature. The results showed significant differences between the groups. Thus, the null hypothesis was rejected.

In the present study, there was a significant difference between the control and irrigation groups. It can be claimed that the saline solution provided a heat sink for more fatigue resistance of the K3XF files during cyclic fatigue tests. Shen et al^12^ investigated the cyclic fatigue resistance of K3XF files in a dry condition and deionized water and concluded that K3XF files’ cyclic fatigue resistance was higher in water than in air. Shen et al^13^ showed that files produced from controlled memory wire were more resistant to cyclic fatigue in liquid than in dry conditions, consistent with the present study.

Besides, there was a significant difference between the irrigation groups. The saline solution at +4°C might provide the temperature to rise later. Thus, K3XF instruments’ fracture associated with cyclic fatigue might be delayed. The data support that more martensitic alloys are more flexible, and therefore more martensitic files are more resistant to fatigue.^16,17^ Martensitic phase renders crack initiation more difficult due to the presence of a larger number of interfaces.^21^ Shen et al^22^ found that the austenite finish temperature of K3XF was 24.89±1.98°C using differential scanning calorimetry analyses. At the above temperature, K3XF instruments are completely austenite. Continuous irrigation with saline solution at room temperature or +4°C might delay austenite transformation. Irrigation with saline solution at +4°C might also provide a more martensitic instrument than at room temperature. This might also explain the significant differences between the groups.

According to our literature search, there is no study on the effect of irrigation solution’s temperature on the cyclic fatigue life. Therefore, a direct comparison between studies is not possible. Grande et al^23^ investigated the effect of a tetrafluoroethane-based cooling spray on cyclic fatigue and reported that this application increases NiTi endodontic files’ flexural fatigue resistance. Grande et al^23^ did not simulate the body temperature; however, they showed that cooling the instrument affected the cyclic fatigue. In the present study, the body temperature was simulated, and the results are consistent with the study by Grande et al.^23^

There was no significant difference between the groups in terms of the fractured fragments’ length in the present study. The files were rotated within a tapered artificial, stainless steel canal with a coronal and an apical curvature (double curve) for the cyclic fatigue resistance test. The coronal curvature’s angle was 60°, and its radius was 5 mm. The deepest point of the coronal curvature was located at 12 mm from the tip of the instrument. The apical curvature’s angle was 70°, and its radius was 2 mm. The deepest point of the apical curvature was located at 7 mm from the tip of the instrument. The fractured fragment’s length of each file was at the center of the coronal curvature (approximately 12 mm from the tip of the instrument). The fact that the groups exhibited similar results in terms of the broken instruments’ length can be caused by the fact that the fracture occurs mainly at the center of the curvature, as reported in previous studies.^8,24^ Yılmaz et al^25^ reported that the curvature center’s location was 5 mm from the apical portion and the fractured instruments’ lengths were similar in all the groups (approximately 5 mm), consistent with the present study.

Many studies are available on the cyclic fatigue life of various files. The majority of these studies have been performed at room temperature, but this does not reflect clinical conditions since the in vivo intracanal temperature is approximately 35°C.^26^ In recent studies, body temperature was simulated, and cyclic fatigue tests were performed at body temperature (37°C). de Vasconcelos et al^18^ reported that increasing the temperature to 37°C, simulating the body temperature, decreased the fracture resistance compared with room temperature (20°C). Dosanjh et al^27^ evaluated the impact of different temperatures (3°C, 22°C, 37°C, and 60°C) on cyclic fatigue and found a significant effect. According to these studies’ results, it might be concluded that cyclic fatigue resistance experiments should be performed at body temperature. In these studies, a water bath was used to simulate body temperature. A similar method was used in the present study, and the body temperature was simulated. This method might yield more appropriate results similar to clinical conditions.^18^

## Conclusion

Within this study’s limitations, continuous irrigation with saline solution increased NiTi instruments’ NCF, and decreasing the saline solution’s temperature increased this effect.

## Authors’ contributions

HA: Planning, materials and methods stage, writing stage, critical revision. EDY: Planning, materials and methods stage, writing stage, critical revision. GT: Materials and methods stage. EK:Materials and methods stage. ET:Materials and methods stage. All the authors have read and agreed to the published version of the manuscript.

## Acknowledgments

Not applicable.

## Competing Interests

The authors declare no competing interests with regards to the authorship and/or publication of this article.

## Ethics Approval

Not applicable.

## Funding

No funding.

## References

[1] Kosa DA, Marshall G, Baumgartner JC (1999). An analysis of canal centering using mechanical instrumentation techniques. J Endod.

[2] Parashos P, Gordon I, Messer HH (2004). Factors influencing defects of rotary nickel-titanium endodontic instruments after clinical use. J Endod.

[3] Sattapan B, Nervo GJ, Palamara JE, Messer HH (2000). Defects in rotary nickel-titanium files after clinical use. J Endod.

[4] Shen Y, Cheung GS, Peng B, Haapasalo M (2009). Defects in nickel-titanium instruments after clinical use. Part 2: Fractographic analysis of fractured surface in a cohort study. J Endod.

[5] Berutti E, Paolino DS, Chiandussi G, Alovisi M, Cantatore G, Castellucci A (2012). Root canal anatomy preservation of WaveOne reciprocating files with or without glide path. J Endod.

[6] Varela-Patino P, Ibanez-Parraga A, Rivas-Mundina B, Cantatore G, Otero XL, Martin-Biedma B (2010). Alternating versus continuous rotation: a comparative study of the effect on instrument life. J Endod.

[7] Shen Y, Haapasalo M, Cheung GS, Peng B (2009). Defects in nickel-titanium instruments after clinical use. Part 1: Relationship between observed imperfections and factors leading to such defects in a cohort study. J Endod.

[8] Gambarini G, Grande NM, Plotino G, Somma F, Garala M, De Luca M (2008). Fatigue resistance of engine-driven rotary nickel-titanium instruments produced by new manufacturing methods. J Endod.

[9] Gutmann JL, Gao Y (2012). Alteration in the inherent metallic and surface properties of nickel-titanium root canal instruments to enhance performance, durability and safety: a focused review. Int Endod J.

[10] Rodrigues CT, Duarte MA, de Almeida MM, de Andrade FB, Bernardineli N (2016). Efficacy of CM-Wire, M-Wire, and Nickel-Titanium instruments for removing filling material from curved root canals: a micro-computed tomography study. J Endod.

[11] Hou X, Yahata Y, Hayashi Y, Ebihara A, Hanawa T, Suda H (2011). Phase transformation behaviour and bending property of twisted nickel-titanium endodontic instruments. Int Endod J.

[12] Shen Y, Zhou H, Campbell L, Wang Z, Wang R, Du T (2014). Fatigue and nanomechanical properties of K3XF nickel-titanium instruments. Int Endod J.

[13] Shen Y, Qian W, Abtin H, Gao Y, Haapasalo M (2012). Effect of environment on fatigue failure of controlled memory wire nickel-titanium rotary instruments. J Endod.

[14] Cheung GS, Shen Y, Darvell BW (2007). Does electropolishing improve the low-cycle fatigue behavior of a nickel-titanium rotary instrument in hypochlorite?. J Endod.

[15] Miyai K, Ebihara A, Hayashi Y, Doi H, Suda H, Yoneyama T (2006). Influence of phase transformation on the torsional and bending properties of nickel-titanium rotary endodontic instruments. Int Endod J.

[16] Santoro M, Nicolay OF, Cangialosi TJ (2001). Pseudoelasticity and thermoelasticity of nickel-titanium alloys: a clinically oriented review. Part I: Temperature transitional ranges. Am J Orthod Dentofacial Orthop.

[17] Nguyen HH, Fong H, Paranjpe A, Flake NM, Johnson JD, Peters OA (2014). Evaluation of the resistance to cyclic fatigue among ProTaper Next, ProTaper Universal, and Vortex Blue rotary instruments. J Endod.

[18] de Vasconcelos RA, Murphy S, Carvalho CA, Govindjee RG, Govindjee S, Peters OA (2016). Evidence for reduced fatigue resistance of contemporary rotary instruments exposed to body temperature. J Endod.

[19] Dauskardt RH, Duerig TW, Ritchie RO. Effect of in situ phase transformation on fatigue-crack propagation in Ti-Ni shape memory alloy. In: Otsuka K, Shimizu K, editors. International Meeting on Advanced Materials. Pittsburgh, PA, USA; 1989: 243-49.

[20] Shen Y, Cheung GS, Bian Z, Peng B (2006). Comparison of defects in ProFile and ProTaper systems after clinical use. J Endod.

[21] Shen Y, Zhou HM, Zheng YF, Peng B, Haapasalo M (2013). Current challenges and concepts of the thermomechanical treatment of nickel-titanium instruments. J Endod.

[22] Shen Y, Zhou HM, Wang Z, Campbell L, Zheng YF, Haapasalo M (2013). Phase transformation behavior and mechanical properties of thermomechanically treated K3XF nickel-titanium instruments. J Endod.

[23] Grande NM, Plotino G, Silla E, Pedulla E, DeDeus G, Gambarini G (2017). Environmental temperature drastically affects flexural fatigue resistance of nickel-titanium rotary files. J Endod.

[24] Grande NM, Plotino G, Pecci R, Bedini R, Malagnino VA, Somma F (2006). Cyclic fatigue resistance and three-dimensional analysis of instruments from two nickel-titanium rotary systems. Int Endod J.

[25] Yilmaz K, Uslu G, Gundogar M, Ozyurek T, Grande NM, Plotino G (2018). Cyclic fatigue resistances of several nickel-titanium glide path rotary and reciprocating instruments at body temperature. Int Endod J.

[26] de Hemptinne F, Slaus G, Vandendael M, Jacquet W, De Moor RJ, Bottenberg P (2015). In vivo intracanal temperature evolution during endodontic treatment after the injection of room temperature or preheated sodium hypochlorite. J Endod.

[27] Dosanjh A, Paurazas S, Askar M (2017). The effect of temperature on cyclic fatigue of nickel-titanium rotary endodontic instruments. J Endod.

